# Genome-Wide Association Analysis of Growth Curve Parameters in Chinese Simmental Beef Cattle

**DOI:** 10.3390/ani11010192

**Published:** 2021-01-15

**Authors:** Xinghai Duan, Bingxing An, Lili Du, Tianpeng Chang, Mang Liang, Bai-Gao Yang, Lingyang Xu, Lupei Zhang, Junya Li, Guangxin E, Huijiang Gao

**Affiliations:** 1 Institute of Animal Science, Chinese Academy of Agricultural Sciences, Beijing 100193, China; xhduan0411@163.com (X.D.); ABX2HF@126.com (B.A.); dulili1996@126.com (L.D.); changtianpeng@126.com (T.C.); liangmang87@163.com (M.L.); xulingyang@163.com (L.X.); zhanglupei@caas.cn (L.Z.); lijunya@caas.cn (J.L.); 2 College of Animal Science and Technology, Southwest University, Chongqing 400715, China; yangbaigao915@163.com

**Keywords:** longitudinal data, growth curve model, single-trait GWAS, multi-trait GWAS, Chinese Simmental beef cattle

## Abstract

**Simple Summary:**

Complex traits that require observations over multiple time points for the same individual are called longitudinal traits. Understanding the genetic architecture of beef cattle growth cannot be limited simply to a genome-wide association study (GWAS) for body weight at any specific ages, but should be extended to a more general purpose by considering the longitudinal weight–age using a growth curve approach. We compared three nonlinear models that described the body weight data of Chinese Simmental beef cattle. The parameters of the suitable model were treated as phenotypes of single-trait GWAS and multi-trait GWAS. We identified 87 significant single nucleotide polymorphisms (SNPs) associated with body weight. Many candidate genes associated with body weight were identified which may be useful for exploring the full genetic architecture underlying growth and development traits in livestock.

**Abstract:**

The objective of the present study was to perform a genome-wide association study (GWAS) for growth curve parameters using nonlinear models that fit original weight–age records. In this study, data from 808 Chinese Simmental beef cattle that were weighed at 0, 6, 12, and 18 months of age were used to fit the growth curve. The Gompertz model showed the highest coefficient of determination (R^2^ = 0.954). The parameters’ mature body weight (A), time-scale parameter (b), and maturity rate (K) were treated as phenotypes for single-trait GWAS and multi-trait GWAS. In total, 9, 49, and 7 significant SNPs associated with A, b, and K were identified by single-trait GWAS; 22 significant single nucleotide polymorphisms (SNPs) were identified by multi-trait GWAS. Among them, we observed several candidate genes, including *PLIN3*, *KCNS3*, *TMCO1*, *PRKAG3*, *ANGPTL2*, *IGF-1*, *SHISA9*, and *STK3*, which were previously reported to associate with growth and development. Further research for these candidate genes may be useful for exploring the full genetic architecture underlying growth and development traits in livestock.

## 1. Introduction

With improved quality of life in China, the demand for meat, particularly beef, is increasing [1]. The Simmental breed accounts for more than 70% of the total number of beef cattle in China [2]. Therefore, it is necessary to analyze the genetic mechanism of the growth traits in beef cattle production [3]. 

Genome-wide association study (GWAS) and genomic selection (GS) are powerful statistical tools that can broadly identify candidate genes with significant single nucleotide polymorphisms (SNPs) involved in production traits [4,5], growth traits [6,7] and fertility traits [8,9]. However, current research mainly focuses on single data records, such as birth weight, weaning weight, and weight before slaughter [3,10,11]. Complex traits that require observations over multiple time points for the same individual are called longitudinal traits. Compared with single data records, longitudinal data can better describe the growth and production of livestock and poultry [12,13]. The fitting growth curve model is one of the most common strategies for such data [14]. Different models [15] provide a few parameters for people to show the regularity of weight gain in livestock, such as mature body weight (A), time-scale parameter (b), and maturity rate (K), which might reflect the influence of genetic impacts on body weight. In the current study, parameters (such as A and K) were considered as phenotypes of the mixed linear model, and quantitative trait loci affecting the growth curve were identified by GWAS. In addition, Das et al. [16] proposed a series of methods based on random regression models. Previous research has demonstrated that these methods are more sophisticated and flexible [17], but they did not provide biologically-interpretable parameters, such as A and K, which are usually required in daily breeding management. 

Generally, a quantitative trait locus (QTL), which affects complex traits, may affect multiple traits [18]. Therefore, the genetic correlations between the parameter estimates (mainly A and K) should be considered. These correlations may be attributed to SNPs that have pleiotropic effects on multiple traits. Therefore, multiple trait GWAS (multi-trait GWAS) is more reasonable in this study [18,19].

In this study, body weights of 808 Chinese Simmental beef cattle at four stages of growth were used to fit the growth curve. The best fitting growth curve parameters were treated as phenotypes for single-trait GWAS and multi-trait GWAS. The aim of our study was to comprehensively analyze candidate genes and QTL regions associated with growth traits by two GWAS methods. We also undertook post-GWAS analyses to identify and prioritize annotated genes within detected genomic regions using the Gene Ontology (GO) and Kyoto Encyclopedia of Genes and Genomes (KEGG) pathways. Our findings offer valuable insights for exploring the full genetic architecture underlying growth and development traits in livestock.

## 2. Materials and Methods

### 2.1. Resource Population and Phenotypes Collection

All animals used in the study were treated following the guidelines established by the Council of China Animal Welfare. Protocols of the experiments were approved by the Science Research Department of the Institute of Animal Sciences, Chinese Academy of Agricultural Sciences (CAAS), Beijing, China (approval number: RNL09/07). The training population consisted of 808 Chinese Simmental beef cattle established in Ulgai, Xilingole League, Inner Mongolia of China. Body weight at four growth stages (0, 6, 12, and 18 months after birth) were measured for each individual. Since fixed effects were related to body weight and not to growth curve parameters, original body weight data at each age were pre-adjusted for fixed effects (breed, year, and month of birth) by a generalized linear model (GLM). The descriptive statistics of the pre-adjusted phenotypic data are presented in Table 1.

### 2.2. Genotyping and Quality Control

Genomic DNA was isolated from blood samples using the TIANamp Blood DNA Kit (Tiangen Biotech Co.Ltd., Beijing, China) and DNA quality was acceptable when the A260/A280 ratio was between 1.8 and 2.0. In total, 808 cattle were genotyped using Illumina BovineHD Beadchip (Illumina Inc., San Diego, CA, USA). Before statistical analysis, SNPs were pre-processed by PLINK v1.07 [20]. Duplicated SNPs were also removed. Finally, 671,192 SNPs on 29 autosomal chromosomes with an average distance of 3 kb were generated for the analysis. SNPs were deleted according to the following standards, including minor allele frequency (<0.01), SNP call rate (<0.05), and Hardy–Weinberg equilibrium values (*p* < 1 × 10^−6^).

### 2.3. Growth Curve Fitting

Three of the most widely used nonlinear models (Table 2) to describe animal growth curves—Gompertz, Logistic, and Brody—were fitted for each animal using the iterative nonlinear least-squares method via the Gauss–Newton [21] algorithm implemented in SAS 9.4. In the function, A is the mature body weight, which means the ultimate body weight of an individual; b is the time-scale parameter, which means the time for an individual to reach its maximum growth rate; K is the maturity rate, which means the rate at which an individual approaches its mature body weight (A).

The coefficient of determination R^2^ [22] was used to evaluate the fitting effect of the growth curve model. The expression is as follows:

(1) $$R^{2}\;=\;1\;-\;\sum \frac{\left(W-\hat{W}\right)}{\left(W-\bar{W}\right)}$$

where *W* represents the observation of body weight, $\hat{W}$ represents the estimated body weight of the growth curve model, and $\bar{W}$ is the average value of body weight.

The genetic correlation of A, b, and K was also calculated, which used the following formula:

(2) $$r_{g}=\frac{\sigma_{a12}}{\sqrt{\;\sigma_{a1}^{2}\sigma_{a2}^{2}\;}}$$

where $\sigma_{a1}^{2}$ is the additive genetic variance of trait 1, $\sigma_{a2}^{2}$ is the additive genetic variance of trait 2, and $\sigma_{a12}$ is the covariance of additive effects.

### 2.4. Single-Trait GWAS

After selecting the nonlinear model which best fit the body weight data, the parameters A, b, and K were used in GWAS. Firstly, we used principal component analysis (PCA) and obtained the kinship matrix using the package GAPIT [23] (Genomic Association and Prediction Integrated Tool) (http://www.maizegenetics.net/gapit) in R software (R 3.6.1). The following mixed linear model was considered:

*y* = *Ws* + *Xβ* + *Zμ* + *e* (3)

where *y* represents the vector of observations from the three phenotypes (A, b, K estimates) for each individual; *s* is the SNP effects vector; *W* is a matrix of SNP genotype indicators, which were coded as 0, 1, and 2 corresponding to the three genotypes AA, AB, and BB; *μ* is a vector of these polygenic effects with an assumed *N* (*0*, *Kσ*^2^) distribution, where *σ^2^* is the polygenic variance and *K* is a marker inferred kinship matrix; *X* is an incidence matrix for *β*, and *β* is a non-genetic vector of fixed effects only including principal component effects (the top three eigenvectors of the Q matrix). The other fixed effects were not included at this step, since they were already considered before fitting nonlinear functions (see pre-adjustment for fixed effects in Section 2.1). *Z* is an incidence matrix for *μ*; while *e* is a vector for random residual errors with a putative *N* (*0,* ${I\sigma }_{e}^{2}$) distribution, where $\sigma_{e}^{2}$ is the residual variance.

The false discovery rate (FDR) was used to determine the threshold values for single-trait GWAS and multi-trait GWAS. The FDR was set as 0.05, and the threshold *p*-value was calculated as follows:

*p* = FDR × n/m (4)

where n is the number of *p* < 0.05 in the results and m is the total number of SNPs [24].

### 2.5. Multi-Trait GWAS

The multi-trait GWAS was conducted to detect pleiotropic SNPs for the parameters A, b, and K. The model was a Chi-squared distribution which was calculated for each SNP using the following formula [25]:

(5) $$t_{i}=\frac{\left|\hat{v_{i}}\right|}{\sqrt{V\left(\hat{v_{i}}\right)}}$$

(6) $$x_{multi-trait}^{2}=t_{i}^{’}V^{-1}t_{i}$$

where $t_{i}$ is a 3 × 1 vector of the t-values for *i*th SNP obtained from single-trait GWAS; ${\hat{v}}_{i}$ is the estimate of *v*; $V\left({\hat{v}}_{i}\right)$ is the corresponding variance which can be obtained by the compressed mixed linear model (CMLM); $t_{i}^{'}$ is the transpose of the vector $t_{i}$; $V^{-1}$ is the inverse of the 3 × 3 correlation matrix between traits, which was calculated using the qualified SNPs.

### 2.6. Gene Function Annotation

We explored the biological mechanism of significant SNPs based on the interpretability of the gene functions related to the relevant SNPs. To select the candidate genes based on the physical location of SNPs, the BioMart module of Ensembl was used to match the significant SNPs with the UMD Bostaurus 3.1 (http://www.animalgenome.org). Then we confirmed the biological function of related genes by the genome databanks National Center for Biotechnology Information (NCBI) (https://www.ncbi.nlm.nih.gov/), and the genes associated with growth and development traits were screened out. GO and KEGG pathways were used to annotate the main biological functions, metabolic pathways, and signal transduction pathways involved in differentially expressed genes.

## 3. Results

### 3.1. Growth Curve Fitting

**Figure 1:**
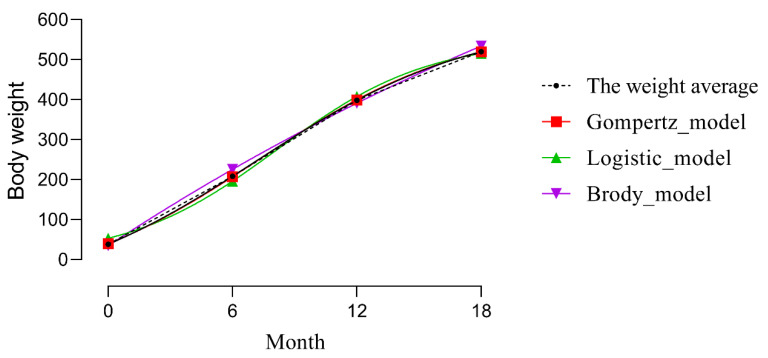
Plot of growth curve models.

Models are shown in Table 3, and the three growth curves are plotted in Figure 1. The R^2^ values for the Gompertz, Logistic, and Brody models were 0.954, 0.951, and 0.951, respectively; the Gompertz model showed the best goodness of fit. Figure 1 shows four growth curves of the Gompertz model, Logistic model, Brody model, and the weight average. The curves representing the Gompertz model and average body weight overlap almost completely, while the other curves have some deviation. Therefore, the parameters of the Gompertz model were selected as phenotypes of GWAS.

The correlation coefficients were 0.087 (A and b), –0.578 (A and K), and 0.369 (b and K) respectively, which showed that A and K have a strong negative correlation.

### 3.2. Principal Components Analysis (PCA)

**Figure 2:**
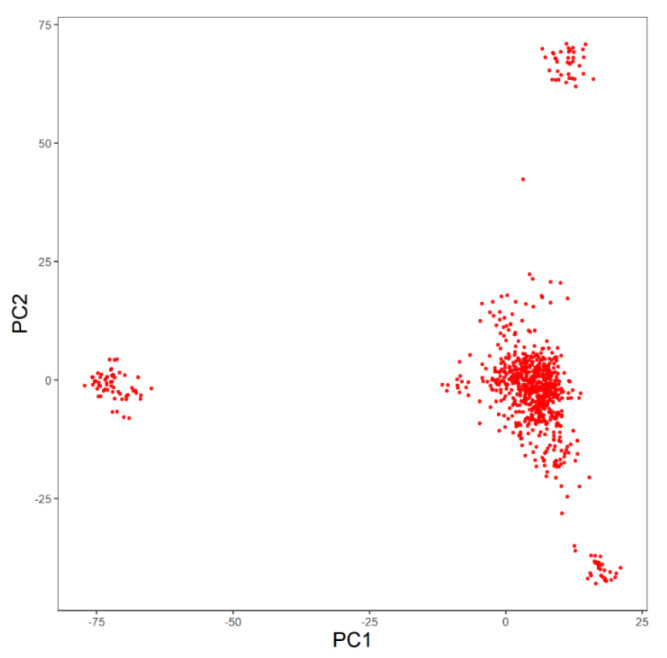
Population structure identified by principal components analysis. PC stands for the principal components of principal components analysis.

The population stratification of the Simmental beef cattle population based on the PCA is shown in Figure 2. The population was divided into five separate clusters, demonstrating an obvious stratification in the reference population. The majority of individuals are located in the lower right corner, while a small number of individuals are distributed in other regions. Therefore, the first two principal components are selected as covariables to eliminate the influence of population stratification on correlation analysis.

### 3.3. Summary of Results by Two GWAS Methods

**Figure 3:**
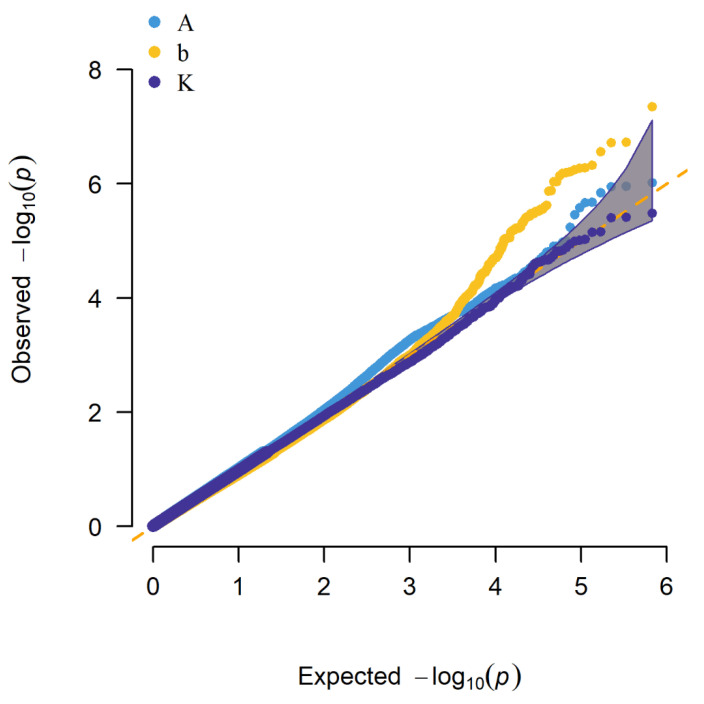
Quantile-quantile (Q-Q) plots of single-trait genome-wide association study (GWAS) for A, b, and K.

**Figure 4:**
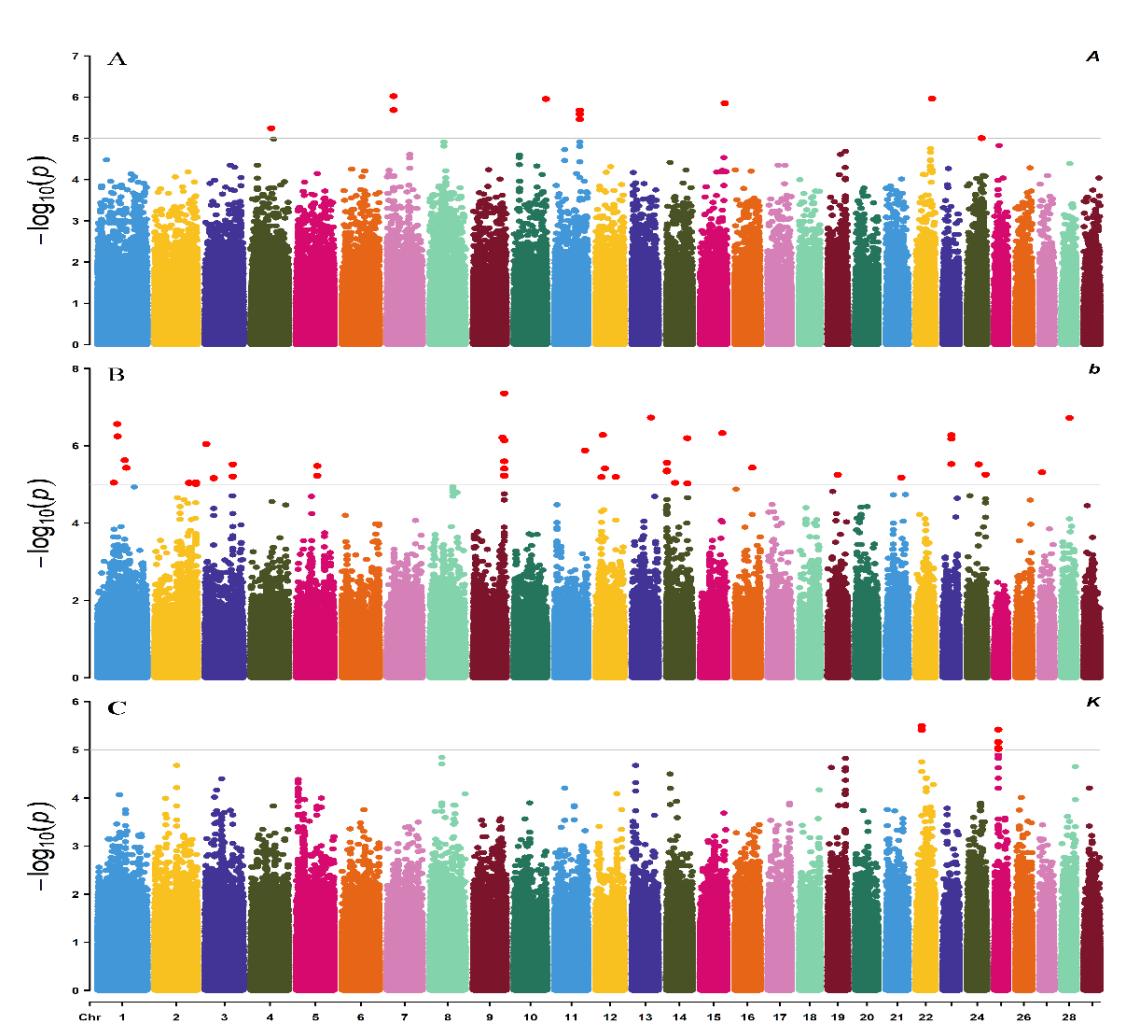
Manhattan plots of GWAS for A, b, and K. (**A**) stands for the Manhattan plot of parameter A; (**B**) stands for the Manhattan plot of parameter b; (**C**) stands for the Manhattan plot of parameter K.

The quantile-quantile (Q-Q) plots and Manhattan plots of single-trait GWAS are shown in Figure 3 and Figure 4. Most points were near the diagonal line in quantile-quantile (Q-Q) plots because the population structure was considered in the GWAS function. The Q-Q plots suggested that there was no inflation or systematic bias in this research. There were nine significant SNPs for mature body weight (A) in the Manhattan plots of single-trait GWAS. The nine SNPs were located on bos taurus autosomes (BTA) 4, 7, 10, 11, 15, and 22, and the locus with the lowest *p*-value was located at 20,500,709 bp on BTA 7 (Figure 4A). The 49 significant SNPs were shown for time-scale parameter (b), and the SNP with the lowest *p*-value was located at 98,989,710 bp on BTA 9. For the maturity rate (K), Manhattan plot indicated seven significant loci which were located on BTA 22 and 25, and the SNP with the lowest *p*-value was located at 18,694,612 bp on BTA 22. We observed several associated genes involved in growth and development, including *PLIN3*, *KCNS3*, *TMCO1*, *ANGPTL2*, *IGF-1*, *ASPH*, *ALPL*, *GRM7*, and *SHISA9*. All results are shown in Table 4.

**Figure 5:**
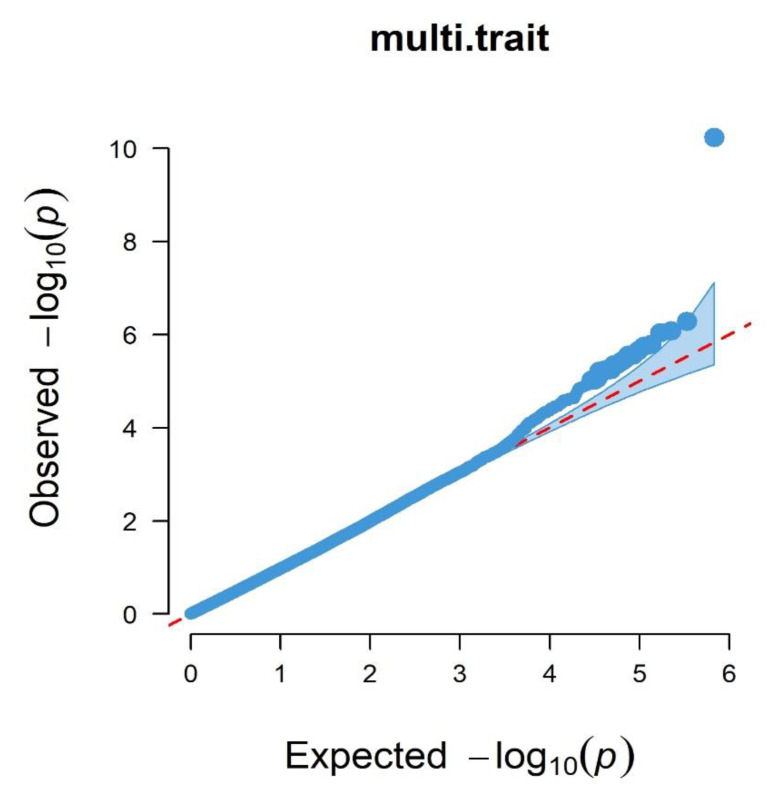
Q-Q plots of multi-trait GWAS for A, b, and K.

**Figure 6:**
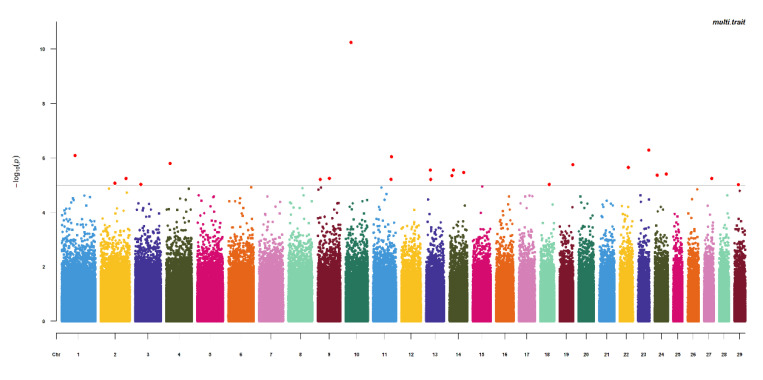
Manhattan plot of multi-trait GWAS for A, b, and K.

The Q-Q plot and the Manhattan plot of multi-trait GWAS are shown in Figure 5 and Figure 6. The same conclusion as the single-trait GWAS was given in the Q-Q plot of multi-trait GWAS. The 22 significant SNPs were identified. The SNP with the lowest *p*-value was located at 25,336,507 bp on BTA 10. We also observed several associated genes involved in growth and development which included *STK3*, *CD58*, and *bta-mir-2285de*. The results are shown in Table 4.

### 3.4. GO and KEGG Pathway Analysis

We found 29 KEGG pathways and 135 GO terms, and 12 pathways and 99 GO terms were significantly enriched (*p* < 0.05) (e.g., thiamine metabolism, circadian rhythm, protein stabilization, nephric duct morphogenesis, glycosylphosphatidylinositol (GPI)-linked ephrin receptor activity) (Table S1). Particularly, seven KEGG pathways and 14 GO terms which were related to growth and development are shown separately in Table 5, including Hippo signaling pathway—multiple species, longevity regulating pathway—multiple species, nephric duct morphogenesis, and limb morphogenesis.

## 4. Discussion

### 4.1. Growth Curve Fitting

R^2^ of Gompertz model reached 0.954, which was the highest of the three models. The parameter A of the Gompertz model showed that the mature body weight of Chinese Simmental beef cattle reached 617.9 kg, which was within the normal mature weight range (600–800 kg) for the population [26]. Though the R^2^ of Logistic model and Brody model reached 0.951, the parameter A (551.0 and 1458.5) was inconsistent with the actual weight of Chinese Simmental beef cattle. The results indicated that the two models may not be suitable for the data in this study. Though the coefficient of determination (R^2^) for Logistic model and Brody model for A are the same (0.951), the estimate of A for the two models was quite different. The reason for this phenomenon may be that the function expressions of the two models are different, and the estimation methods are also different, so the models adapt to different breeds. Among the three models, the growth curve fitting by the Gompertz model with the highest R^2^ had well-matched performance for the actual cattle population. Therefore, the Gompertz model was chosen as the best model for Chinese Simmental beef cattle, which was the same conclusion as Liang et al. [27].

The negative relationship between parameters A and K has been reported many times [14,28,29], which suggests that individuals with smaller mature weight will gain its mature body weight at a young age. Thus, we can predict that precocious animals will not gain a high mature body weight, even if we put in the same cost (such as feed) as other individuals. The conclusion could help us reduce the cost of raising animals by learning to manage individuals separately.

Although there are few studies about growth curves in Chinese Simmental beef cattle, some authors have concluded that the Gompertz model provides the best fit for body weight of beef cattle. Zainaguli et al. [30] used four common models (Logistic, Gompertz, Brody, and Bertallanffy) to fit the weight growth curves of 344 Xinjiang Brown cattle. The Gompertz model showed the best fit for the population. Liang et al. [27] compared four growth curve models (Logistic, Gompertz, Brody, and Bertallanffy) fitted to body weight of Simmental beef cattle and concluded that the Gompertz model was superior to the other models.

### 4.2. GWAS, GO, and KEGG Pathway Analysis

We performed single-trait GWAS and multi-trait GWAS for the body weight trait of Chinese Simmental beef cattle. A great number of genes involved in growth and development were identified by each method. The reason for this phenomenon may be the limited dataset. However, since most growth and development traits are controlled by multiple genes [31], the genes associated with growth and development identified by separate GWAS cannot be ignored. Single-trait GWAS and multi-trait GWAS have their specific advantages in the identification of distinct loci. For example, compared to the meta-analysis GWAS, the single-population GWAS was more powerful for the identification of SNPs [32], whereas multi-trait GWAS has the advantage of increasing statistical power and identifying pleiotropic loci [33,34,35]. Therefore, it should be noted that multi-trait GWAS cannot replace single-trait GWAS, rather it was complementary to single-trait GWAS. Thus, combining single-trait GWAS and multi-trait GWAS methods was expected to markedly improve the analysis of the genetic mechanism of the body weight traits for Chinese Simmental beef cattle.

Single-trait GWAS: For mature body weight (A), the significant locus ARS-BFGL-NGS-14531 which has the lowest *p*-value was near *PLIN3* (*perilipin 3*). *PLIN3* is an important regulator of adipogenesis and triglyceride storage [36], and *PLIN3* functions are intertwined with the lipogenic pathways implicated in sebaceous lipogeneses, such as desaturation and triglyceride synthesis [37]; three significant SNPs were near *KCNS3* (*potassium voltage-gated channel modifier subfamily S member 3*) which was proven to be significantly associated with the percent body fat (%BF) [38]. For time-scale parameter (b), two SNPs were within *TMCO1* (*transmembrane and coiled-coil domains 1*), which may affect muscle development because of the significant relationship with *PRKAG3* (*protein kinase AMP-activated non-catalytic subunit gamma 3*); two significant SNPs were near *ANGPTL2* (*angiopoietin like 2*). A study showed that *ANGPTL2* may be used as a new type of adipocyte factor [39]. One SNP was located in *CFB* (*complement factor B*), which has been identified as related to the total number of piglets born (TNB) and reproductive traits [40]; four significant SNPs were near or within *IGF-1* (*insulin-like growth factor 1*). *IGF-1* and its signaling pathway play a primary role in normal growth and aging [41,42]. The locus BovineHD1400008371 was near *ASPH* (*aspartate beta-hydroxylase*), which is involved in regulating the growth and development of beef cattle carcass [43]; four significant SNPs were near or within *ALPL* (*alkaline phosphatase, biomineralization associated*). A study showed that the expression level of *ALPL* in white blood cells of obese people is significantly higher than that of lean people, indicating that *ALPL* may be related to the production of fat [44]; two significant loci were within *EPHA4* (*EPH receptor A4*) which was one of the potential candidate genes for growth trait of pigs [45]. Seven significant loci of maturity rate (K) were concentrated on chromosomes 22 and 25; two associated genes *GRM7* (*glutamate metabotropic receptor 7*) and *SHISA9* (*shisa family member 9*) were found and *SHISA9* was highly correlated with growth and development [46].

Multi-trait GWAS: *CD58* (*CD58 molecule*) and *STK3* (*serine/threonine kinase 3*) were found by both methods. Two SNPs (BovineHD1400018901 and BovineHD1400018902) from single-trait GWAS and one SNP (BovineHD1400018913) from multi-trait GWAS were near *STK3*, which is also named *MST2* (*macrophage stimulating 2*). *MST1* (*macrophage stimulating 1*) and *MST2* were central to the Hippo signaling pathway in mammals, which enabled the dynamic regulation of tissue homeostasis in animal development [47]. One significant SNP (BovineHD0100017897) was within *bta-mir-2285de* which might be an important regulator of bovine mammary lipogenesis and metabolism [48]. The locus BovineHD2700010439 was near *KAT6A* (*lysine acetyltransferase 6A*), and it has been shown to be significantly associated with growth retardation [49]. One significant SNP(BovineHD1100023984) was near *NBAS* (*NBAS subunit of NRZ tethering complex*) which was significantly associated with bone development [50].

GO and KEGG pathway: There are also some candidate genes which are closely related to growth and development found by GO and KEGG pathway. For example, *STK3* was involved in more than one GO and KEGG pathway, including Hippo signaling pathway—multiple species, Hippo signaling pathway, MAPK signaling pathway, cell differentiation involved in embryonic placenta development [51], hepatocyte apoptotic process, negative regulation of organ growth, negative regulation of cell population proliferation, positive regulation of fat cell differentiation, and central nervous system development, which suggests that *STK3* may be closely related to cell proliferation and differentiation [51], organ growth and development, and nervous system development [52]. *ASPH* was involved in limb morphogenesis and roof of mouth development, which suggests that *ASPH* is significantly associated with body development [43]. *ANGPTL2* was involved in angiogenesis, which suggests that *ANGPTL2* is closely related to the development of individuals [39].

## 5. Conclusions

In conclusion, the three growth curve models were used to fit the body weight data of Chinese Simmental beef cattle. The parameters of the Gompertz model with the best fitting effect are the phenotypes of GWAS. A total of 65 significant SNPs from single-trait GWAS and 22 SNPs from multi-trait GWAS were found. Seven KEGG pathways and 14 GO terms, which were related to growth and development, were also identified. Several candidate genes that were significantly associated with growth and development traits were observed, including *PLIN3, KCNS3, TMCO1, ANGPTL2, CFB, IGF-1, ALPL EPHA4, SHISA9, STK3*, and *bta-mir-2285de*. The role of associated genes in growth and development was also discussed. Further research for these candidate genes may be useful for exploring the full genetic architecture underlying growth and development traits in livestock.

## Figures and Tables

**Table 1:** The descriptive statistics of body weight for Chinese Simmental beef cattle.

Age (Month)	Max (kg)	Min (kg)	Mean (kg)	Standard Deviation (SD)
0	55.20	25.00	38.79	6.21
6	326.00	107.00	208.68	39.48
12	561.00	242.00	398.70	56.21
18	739.00	346.00	520.10	73.18

**Table 2:** Growth curve model. A is the mature body weight, b is the time-scale parameter, K is the maturity rate, W is the observed body weight, t is the growth time, and e is the natural logarithm.

Model	Function
Gompertz	W = Aexp(−bexp^−Kt^)
Logistic	W = A(1 + bexp^−Kt^)^−1^
Brody	W = A(1 − bexp^−Kt^)

**Table 3:** Estimated values of the growth curve model.

Parameter	Models		
	Gompertz	Logistic	Brody
A	617.900	551.000	1458.500
b	2.740	9.304	0.976
K	0.153	0.273	0.024
R^2^	0.954	0.951	0.951

**Table 4:** The results of single-trait GWAS and multi-trait GWAS.

Trait	SNP	BTA	Position	Distance	Gene	*p*-Value
**A**	ARS-BFGL-NGS-14531	7	20,500,709	6291	*PLIN3*	9.55 × 10^−7^
	BovineHD2200014587	22	51,133,487	within (intronic)	*BSN*	1.10 × 10^−6^
	BovineHD1000029459	10	101,577,026	within (intronic)	*TTC8*	1.12 × 10^−6^
	BovineHD1500022754	15	78,218,321	within (intronic)	*C15H11ofF49*	1.42 × 10^−6^
	BovineHD0700005699	7	20461 012	within (intronic)	*UHRF1*	2.08 × 10^−6^
	BovineHD1100023174	11	80,858,593	14,458	*KCNS3*	2.13 × 10^−6^
	BovineHD1100023180	11	80,883,741	39,606	*KCNS3*	2.61 × 10^−6^
	BovineHD1100023175	11	80,860,546	16,411	*KCNS3*	3.46 × 10^−6^
	Hapmap36353-SCAFFOLD29708_3468	4	64,923,141	62,596	*PDE1C*	5.78 × 10^−6^
**b**	BovineHD0900028514	9	98,989,710	within (exonic)	*PRKN*	4.43 × 10^−8^
	BovineHD1300017399	13	60,669,478	15,189	*RSPO4*	1.86 × 10^−7^
	BTB-00981633	28	24,967,427	within (intronic)	*DNA2*	1.90 × 10^−7^
	BovineHD1500020257	15	70,169,617	1,074,228	*LRRC4C*	4.73 × 10^−7^
	BovineHD1200006711	12	22,401,586	317,236	*COG6*	5.26 × 10^−7^
	BovineHD2300007448	23	27,217,994	within (intronic)	*SKIV2L*	5.33 × 10^−7^
	BovineHD0900026419	9	93,361,299	12,446	*NOX3*	6.17 × 10^−7^
	BovineHD1400018901	14	67,713,519	within (intronic)	*STK3*	6.38 × 10^−7^
	BovineHD2300007441	23	27,195,210	within (intronic)	*C4A*	6.58 × 10^−7^
	BovineHD0900028515	9	98,990,425	within	*PRKN*	7.26 × 10^−7^
	BovineHD0300000940	3	3,186,646	within (intronic)	*TMCO1*	9.11 × 10^−7^
	BovineHD0300000941	3	3,189,462	within (intronic)	*TMCO1*	9.11 × 10^−7^
	BovineHD1100028458	11	97,919,703	60,177	*ANGPTL2*	1.33 × 10^−6^
	BovineHD1100028450	11	97,903,021	43,495	*ANGPTL2*	1.34 × 10^−6^
	BovineHD0100024671	1	86,573,589	93,224	*DNAJC19*	2.37 × 10^−6^
	BovineHD0900028520	9	99,001,573	within (exonic)	*PRKN*	2.54 × 10^−6^
	BovineHD1400000353	14	2,382,595	within (intronic)	*ZC3H3*	2.76 × 10^−6^
	BovineHD1400000354	14	2,384,748	within (intronic)	*ZC3H3*	2.76 × 10^−6^
	BovineHD2300007455	23	27,227,600	within (intronic)	*CFB*	3.00 × 10^−6^
	BovineHD2400010016	24	36,578,137	458,512	*ADCYAP1*	3.03 × 10^−6^
	BovineHD0300025174	3	87,908,532	16,189	*MYSM1*	3.07 × 10^−6^
	BovineHD0500018625	5	66,594,318	within (intronic)	*IGF-1*	3.34 × 10^−6^
	BovineHD0500018629	5	66,609,814	5314	*IGF-1*	3.34 × 10^−6^
	BovineHD0500018633	5	66,624,481	19,981	*IGF-1*	3.34 × 10^−6^
	BovineHD0100026284	1	92,441,255	1,184,964	*NLGN1*	3.72 × 10^−6^
	BovineHD1200008652	12	29,267,967	within (exonic)	*RXFP2*	3.85 × 10^−6^
	BovineHD0900028524	9	99,010,494	within (exonic)	*PRKN*	3.89 × 10^−6^
	BovineHD1400000321	14	2,241,832	6798	*MAPK15*	4.36 × 10^−6^
	BovineHD1400000343	14	2,348,518	3233	*GSDMD*	4.68 × 10^−6^
	BovineHD1900009534	19	32,360,589	within (intronic)	*HS3ST3A1*	5.67 × 10^−6^
	BovineHD0900028481	9	98,914,727	within (exonic)	*PRKN*	5.95 × 10^−6^
	BovineHD0900028509	9	98,984,305	within (exonic)	*PRKN*	5.95 × 10^−6^
	BovineHD0500018642	5	66,654,472	49,972	*IGF-1*	6.01 × 10^−6^
	BovineHD0900028504	9	98,967,507	within (exonic)	*PRKN*	6.05 × 10^−6^
	BovineHD0300025183	3	87,959,712	within (intronic)	*MYSM1*	6.27 × 10^−6^
	BovineHD1200027060	12	64,329,068	1,659,351	*SLITRK5*	6.45 × 10^−6^
	BovineHD1200026793	12	18,310,824	9625	*RCBTB2*	6.50 × 10^−6^
	BovineHD2100014355	21	49,967,674	200,864	*FBXO33*	6.69 × 10^−6^
	BovineHD0300008509	3	26,888,743	28,039	*CD58*	6.84 × 10^−6^
	BovineHD0300008508	3	26,885,838	25,134	*CD58*	6.98 × 10^−6^
	BovineHD0200038336	2	131,809,255	within (intronic)	*ALPL*	8.81 × 10^−6^
	BovineHD0200038337	2	131,810,815	within (exonic)	*ALPL*	8.81 × 10^−6^
	BovineHD0200038343	2	131,820,288	7428	*ALPL*	8.81 × 10^−6^
	BovineHD0200031784	2	110,303,552	within (intronic)	*EPHA4*	9.04 × 10^−6^
	BovineHD0100014672	1	52,227,088	136,783	*CCDC54*	9.06 × 10^−6^
	BovineHD1400008371	14	28,916,088	27,997	*ASPH*	9.15 × 10^−6^
	BovineHD0200031783	2	110,302,531	within (intronic)	*EPHA4*	9.19 × 10^−6^
	BovineHD1400018902	14	67,716,121	within (intronic)	*STK3*	9.42 × 10^−6^
	BovineHD0200038341	2	131,817,068	4208	*ALPL*	9.84 × 10^−6^
**K**	BovineHD2200005378	22	18,694,612	60,070	*GRM7*	3.24 × 10^−6^
	BovineHD2500003405	25	12,148,764	444,406	*SHISA9*	3.82 × 10^−6^
	BovineHD2200005379	22	18,697,043	57,639	*GRM7*	3.89 × 10^−6^
	BovineHD2500003397	25	12,122,951	418,593	*SHISA9*	6.89 × 10^−6^
	BovineHD2500003394	25	12,119,907	415,549	*SHISA9*	7.09 × 10^−6^
	BovineHD2500003411	25	12,164,708	460,350	*SHISA9*	9.25 × 10^−6^
	BovineHD2500003396	25	12,122,067	417,709	*SHISA9*	9.70 × 10^−6^
**Multi**	BovineHD1000008269	10	25,336,507	11,871	*BT.86117*	5.76 × 10^−11^
	BovineHD2300014561	23	49,948,237	785	*C6ORF146*	5.11 × 10^−7^
	BovineHD0100017897	1	63,214,855	within (intronic)	*bta-mir-2285de*	8.19 × 10^−7^
	BovineHD1100024571	11	85,545,380	311,744	*TRIB2*	9.06 × 10^−7^
	BovineHD1900017810	19	61,961,078	within (intronic)	*ABVA10*	1.78 × 10^−6^
	BovineHD2200011596	22	40,545,626	185,054	*BT.92027*	2.22 × 10^−6^
	BovineHD1300005737	13	19,728,845	183,012	*NRP1*	2.77 × 10^−6^
	BovineHD1400005409	14	18,830,773	441,600	*BT.88023*	2.77 × 10^−6^
	BovineHD1400018913	14	67,761,416	within (exonic)	*STK3*	3.41 × 10^−6^
	BovineHD2400015566	24	54,582,317	107,987	*C18ORF26*	3.89 × 10^−6^
	Hapmap46842-BTA-57397	24	11,851,627	637,616	*CDH7*	4.32 × 10^−6^
	BovineHD1400003514	14	12,051,695	146,289	*GSDMC*	4.44 × 10^−6^
	BovineHD0200034312	2	118,915,870	within (intronic)	*PSMD1*	5.65 × 10^−6^
	BovineHD0900014829	9	53,862,308	48,559	*GPR63*	5.70 × 10^−6^
	BovineHD2700010439	27	36,460,835	72,147	*KAT6A*	5.71 × 10^−6^
	BovineHD1100023984	11	83,388,941	200,644	*NBAS*	6.15 × 10^−6^
	BovineHD0900002818	9	11,192,144	488,896	*RIMS1*	6.15 × 10^−6^
	BovineHD1300006393	13	21,900,826	within (intronic)	*PLXDC2*	6.15 × 10^−6^
	BovineHD0200019309	2	66,721,486	808,880	*ACTR3*	8.50 × 10^−6^
	BovineHD1800012623	18	42,743,057	295,489	*ZNF507*	9.33 × 10^−6^
	BovineHD0300008523	3	26,920,280	59,576	*CD58*	9.36 × 10^−6^
	BovineHD2900005573	29	19,238,772	49,975	*GDPD4*	9.53 × 10^−6^

**Table 5:** The Kyoto Encyclopedia of Genes and Genomes (KEGG) pathways and Gene Ontology (GO) involved in differentially expressed genes.

Gene Name	Term	Database	ID	DEG
*ALPL*	Hippo signaling pathway—multiple species	KEGG pathway	bta00730	1
	biomineral tissue development	Gene Ontology	GO:0031214	1
*ANGPTL2*	angiogenesis	Gene Ontology	GO:0001525	1
*EPHA4*	Axon guidance	KEGG pathway	bta04360	1
	nephric duct morphogenesis	Gene Ontology	GO:0072178	1
	cochlea development	Gene Ontology	GO:0090102	1
*KAT6A*	Signaling pathways regulating pluripotency of stem cells	KEGG pathway	bta04550	1
*PLIN3*	lipid storage	Gene Ontology	GO:0019915	1
*PRKAG3*	Longevity regulating pathway—multiple species	KEGG pathway	bta04213	1
	Apelin signaling pathway	KEGG pathway	bta04371	1
	fatty acid biosynthetic process	Gene Ontology	GO:0006633	1
*ASPH*	limb morphogenesis	Gene Ontology	GO:0035108	1
	roof of mouth development	Gene Ontology	GO:0060021	1
*ASPH, STK3*	negative regulation of cell population proliferation	Gene Ontology	GO:0008285	2
*STK3*	Hippo signaling pathway	KEGG pathway	bta04390	1
	MAPK signaling pathway	KEGG pathway	bta04010	1
	cell differentiation involved in embryonic placenta development	Gene Ontology	GO:0060706	1
	hepatocyte apoptotic process	Gene Ontology	GO:0097284	1
	negative regulation of organ growth	Gene Ontology	GO:0046621	1
	positive regulation of fat cell differentiation	Gene Ontology	GO:0045600	1
	central nervous system development	Gene Ontology	GO:0007417	1

Note: DEG represents the number of differentially expressed genes detected in this pathway; MAPK represents the mitogen-activated protein kinase signaling pathway.

## Data Availability

We confirm that all raw data underlying our findings are publicly available without restriction. Data is available from the Dryad Digital Repository: doi: https://doi.org/10.5061/dryad.4qc06.

## Supplementary Materials

The following are available online at https://www.mdpi.com/2076-2615/11/1/192/s1, Table S1: Gene Ontology (GO) and KEGG pathway analysis.

## Abbreviations

The following abbreviations are used in this manuscript:

GWAS	genome-wide association study
GS	genomic selection
SNP	single nucleotide polymorphism
QTL	quantitative trait loci
FDR	false discovery rate
PCA	principal components analysis
BTA	Bos taurus autosomes
